# Transcriptomic analysis of asymptomatic and symptomatic severe Turkish patients in SARS-CoV-2 infection

**DOI:** 10.14744/nci.2022.28000

**Published:** 2022-04-13

**Authors:** Sadrettin Pence, Burcu Caykara, Halime Hanim Pence, Saban Tekin, Birsen Cevher Keskin, Ali Tevfik Uncu, Ayse Ozgur Uncu, Erman Ozturk

**Affiliations:** 1 Department of Physiology, Istanbul Medeniyet University Faculty of Medicine, Istanbul, Turkey; 2 Department of Biochemistry, Health Sciences University Faculty of Medicine, Istanbul, Turkey; 3 Department of Medical Biology, Health Sciences University, Faculty of Medicine, Istanbul, Turkey; 4 TUBITAK, Marmara Research Center, Gene Engineering and Biotechnology Institute, Kocaeli, Turkey; 5 Department of Molecular Biology and Genetics, Necmettin Erbakan University Faculty of Science, Konya, Turkey; 6 Department of Biotechnology, Necmettin Erbakan University Faculty of Science, Konya, Turkey; 7 Division of Hematology, Department of Internal Diseases, Istanbul Medeniyet University Faculty of Medicine, Istanbul, Turkey

**Keywords:** COVID-19, gene ontology, immunity, innate, platelet activation, RNA-Seq

## Abstract

**Objective:**

Coronavirus disease 2019 (COVID-19), leading to mild infection (MI), acute respiratory distress syndrome or death in different persons. Although the basis of these variabilities has not been fully elucidated, some possible findings have been encountered. In the present study, we aimed to reveal genes with different expression profiles by next-generation sequencing of RNA isolated from blood taken from infected patients to reveal molecular causes of different response.

**Methods:**

Two healthy, severe acute respiratory syndrome coronavirus 2 (SARS-CoV-2)-negative control individuals (NCI), two SARS-CoV-2-positive patients who have MI, and two patients who have critical infection (CI) were included in the study. Total RNA was extracted from blood samples and sequenced. Raw RNA-Seq data were analyzed on Galaxy platform for the identification of differentially expressed genes and their pathway involvements.

**Results:**

We found that 199 and 521 genes were downregulated in whole blood of COVID-19-positive CI patients compared to NCI and MI patients, respectively. We identified 21 gene ontology pathways commonly downregulated in CI patients compared to both NCI and MI, mostly associated with innate and adaptive immune responses. Three hundred and fifty-four and 600 genes were found to be upregulated compared to NCI and MI, respectively. Upregulated six pathways included genes that function in inflammatory response and inflammatory cytokine release.

**Conclusion:**

The transcriptional profile of CI patients deviates more significantly from that of MI in terms of the number of differentially expressed genes, implying that genotypic differences may account for the severity of SARS-CoV-2 infection and inflammatory responses through differential regulation of gene expression. Therefore, further studies that involve whole genome analysis coupled with differential expression analysis are required in order to determine the dynamics of genotype – gene expression profile associations.

**O**ne of the main agents of the seasonal cold, human coronaviruses, is known to cause pneumonia. In 2019, a new coronavirus called severe acute respiratory syndrome coronavirus 2 (SARS-CoV-2) causing the coronavirus disease 2019 (COVID-19) emerged, leading to acute respiratory distress syndrome, death, or asymptomatic infection in different persons [1]. SARS-CoV-2 infection is thought to be asymptomatic in at least one-third of the incidences. Approximately 40% of infected individuals remain asymptomatic [2]. In symptomatic cases, about 81% of infected humans have a mild flu-like illness, while severe or critical disease effects are seen about in 14% and 5% of infected humans, respectively. The overall case-fatality rate is estimated to be 2.3% [3]. Advanced age and the comorbidities with obesity, hypertension, or diabetes mellitus may predispose patients to an increased risk of severe disease and death [4, 5].

Although the cause of the differential response to SARS-CoV-2 infection in different patients has not been fully elucidated, some possible findings have been encountered. For instance, a study showed that patients with severe SARS-CoV-2 infection exhibit a higher antibody response to spike and nucleocapsid proteins of SARS-CoV-2, and a higher memory B-cell response to spike protein [6]. In a recent trial, platelet gene expression was differed between control and the critical ill COVID-19 patients which P-selectin expression had increased [7]. Furthermore, upper airway gene expression reveals suppression of innate immune response [8]. The entry of SARS-CoV-2 into the host cell takes place through angiotensin-converting enzyme 2 (ACE2). Variants in certain regions of the ACE2 gene may affect conformational changes and interaction in spike protein binding, protein stability, while some other variants may cause an increase in ligand-receptor affinity. In particular, variations in regulatory and non-coding genomic regions such as the ACE2 promoter may affect ACE2 expression levels, putatively leading to differential response to SARS-CoV-2 in different individuals [9].

In the present work, we aimed to reveal differentially expressed genes in blood from SARS-CoV-2-infected individuals to identify the molecular causes underlying the variation in response to SARS-CoV-2 infection in different individuals.

## Materials and Methods

### Patients and Sample Collection

Ethical approval for the study was obtained from Istanbul Medeniyet University, Goztepe Training and Research Hospital Ethics Committee for Clinical Research (no: 2020/183, 23.03.2020). Two healthy non-infected control individuals (NCI), two SARS-CoV-2-positive patients who have mild infection (MI), and two SARS-CoV-2-positive patients who have critical infection (CI), as described before [10], were included in the study. Patients were treated as recommended by the TC Ministry of Health. Favipravir was administered to both groups, but patients treated in intensive care unit received steroids. Blood samples were collected from patients in DNA/RNA shield tubes through an indwelling intravenous catheter. Samples were transferred to TUBITAK, Marmara Research Center, Gene Engineering and Biotechnology Institute and RNA isolation process was completed within 24 h in biosafety level 3 laboratory.

**Highlight key points**

• COVID-19 infection disease is heterogeneous and not predictable.
• Innate and adaptive immunity were downregulated in CI.
• Platelet activation and platelet degranulation pathways were exclusively upregulated in CI.
• These factors may be the cause of immunothrombosis.

### RNA Isolation and Quality Assessment

Total RNA was extracted from blood samples using Zymo Quick-RNA™ Whole Blood (Zymo Research Corp, Irvine, U.S.A Catalog No. R1201), according to the manufacturer’s instructions with some modifications. For all three patient groups, 300 μl whole blood was used. After the DNase I treatment in column, RNA was eluted from the column with 15 μl RNase-free water. The quality and quantity of resulting total RNA samples were controlled by Nanodrop spectrophotometer (Thermo Scientific™ NanoDrop) and Qubit (Thermo Fischer Qubit™ 4 Fluorometer). The A260/A280 ratio of purified RNAs was between 1.8 and 2.0, and A260/A230 ratio ranged between 1.8 and 2.1. The RNA integrity number (RIN) was assessed using the Bioanalyzer (Agilent Technologies 2100 Bioanalyzer) for all RNA samples. RNA samples with a RIN number >7 were subjected to cDNA library preparation protocol. The concentration of RNA was measured using the RNA-BR protocol of the Qubit 2.0 Fluorometer (Thermo Fischer Life Technologies).

### Transcriptome Sequencing

For the removal of mitochondrial and cytoplasmic rRNA from total RNA samples, Ribo-Zero Gold rRNA depletion protocol (Illumina TruSeq Stranded Total RNA Library Prep Gold kit, Cat No: 20020599) was used. Depleted and purified samples (200 ng RNA) underwent enzymatic fragmentation. After the first and second strand cDNA synthesis, adenylation, adapter ligation, and amplification procedures were performed with some modifications in the TruSeq Stranded Total RNA protocol (TruSeq Stranded Total RNA Library Prep Gold kit Illumina, Cat no. 20020599). For the adapter ligation step, IDT for Illumina TruSeq RNA unique dual indexes (UDIs) was used (Illumina, Cat no. 20022371). Enrichment of the DNA fragments that have adapter sequences on both ends was carried out by 15 cycles of PCR. After the clean-up procedure of the amplified DNA with AMPure XP beads, the size and purity of the libraries were assessed with Bioanalyzer using the HS DNA Kit (Agilent Technologies 2100 Bioanalyzer). To obtain optimum cluster densities and high-quality data, library quantification was performed by qPCR (KAPA Library Quantification Kits KK4601). The quantity of the libraries was controlled using three replications and two dilutions for each sample for accurate quantification. After the calculation, master pool was loaded onto the NovaSeq 6000 platform, using 150 bp paired ends sequencing and S1 flow cell. The quality score of the run (QC30) was 80.

### RNA-Seq Data Analysis

Raw RNA-Seq data were analyzed using Galaxy platform [11]. Trimmomatic tool was used to eliminate short reads and sequencing adapters (Galaxy Version 0.38.0) [12]. Remaining reads were aligned to human reference genome hg38 using STAR (Version 2.7.5b) to map the reads in their locations in the reference genome [13]. Gene model for hg38 was chosen as ensGene and retrieved from UCSC Genome Browser (https://genome.ucsc.edu/cgi-bin/hgGateway). Quantification of mapped transcripts was done by feature counts tool [14]. This step can simply be summarized as the determination of the number of RNA-Seq reads obtained from each gene/mapped genomic location. Following the quantification process, Deseq2 [15] was used for the identification of differentially expressed genes. Functional analysis of DEGs was conducted through David (v6.8) [16]. The threshold for statistical significance was chosen as 0.05 after multiple testing correction.

Differential gene expression patterns of SARS-CoV-2 were obtained by RNA-Seq transcriptome sequencing performed on whole-blood RNA samples of each group. Differentially expressed genes in CI patients with respect to NCI and MI were determined based on a cutoff log2-fold change value of 2. Gene ontology (GO) terms were assigned to the differentially expressed genes and GO enrichment analysis was performed to determine the up- and down-regulated pathways with putative relations to SARS-CoV-2 infection response in CI patients.

## Results

**Figure 1.**
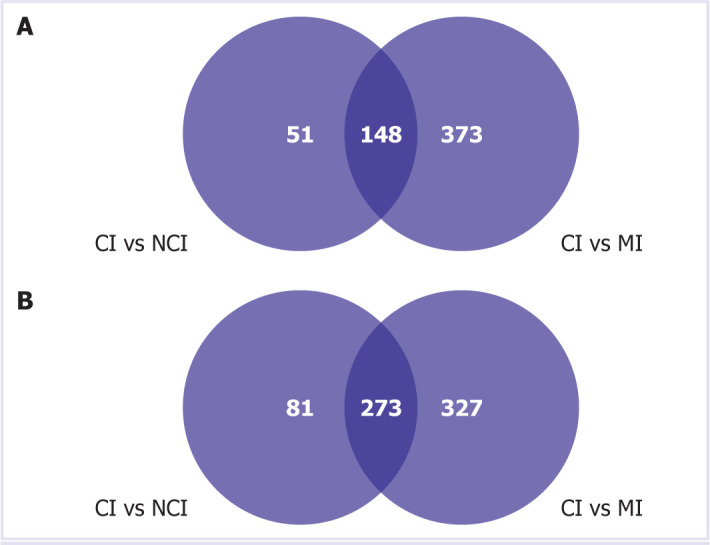
Venn diagrams of differentially expressed gene sets in CI patients. **(A)** Numbers of downregulated genes in CI patients versus NCI and CI versus MI are displayed. **(B)** Numbers of upregulated genes in CI patients versus NCI and CI versus MI are displayed. NCI: SARS-CoV-2-negative control individuals; MI: Mild infection; CI: Critical infection.

Six samples were evaluated in three groups abbreviated as CI, MI, and NCI (Table 1). Patients who have CI died at the intensive care unit. According to the results of the differential expression analysis, 199 and 521 genes were found to be downregulated in whole blood of CI patients compared to NCI and MI, respectively (Table 1), with 148 genes found to be commonly downregulated in both comparisons (Fig. 1a). GO enrichment analysis identified 21 GO pathways commonly downregulated in CI patients with respect to both NCI and MI (Table 2). Majority of these common downregulated GO pathways (17 pathways) were directly associated with innate and adaptive immune responses. Remaining downregulated GO pathways that were not common between the two comparisons (CI vs. NCI and CI vs. MI) were also associated with the immune system responses, including pathways such as innate immune response (GO:0045087), inflammatory response (GO:0006954), B-cell activation (GO:0042113), B-cell receptor signaling (GO:0050853), T-cell differentiation (GO:0030217), and chemokine-mediated signaling (GO:0070098) (Table 2).

**Table 1.** Demographic and clinical characteristics of the patients

Patients	Age	Sex	Groups
ES	25	F	NCI
OE	27	F	NCI
IB	37	F	MI
ST	35	M	MI
AC	76	M	CI
ZY	78	F	CI

F: Female; M: Male; NCI: SARS-CoV-2-negative control individuals; MI: Mild infection; CI: Critical infection.

**Table 2.** The comparison of GO pathways downregulated in CI, MI, and NCI patients

GO pathways downregulated in CI patients	
CI versus NCI*	CI versus MI*
GO:0006955: Immune response	GO:0050776: Regulation of immune response
GO:0007166: Cell surface receptor signaling pathway	GO:0002250: Adaptive immune response
GO:0006968: Cellular defense response	GO:0006955: Immune response
GO:0050776: Regulation of immune response	GO:0006968: Cellular defense response
GO:0031295: T-cell costimulation	GO:0007166: Cell surface receptor signaling pathway
GO:0046641: Positive regulation of alpha-beta-T-cell proliferation	hsa04640: Hematopoietic cell lineage
hsa05340: Primary immunodeficiency	hsa05340: Primary immunodeficiency
hsa04660: T-cell receptor signaling pathway	GO:0042110: T-cell activation
GO:0050852: T-cell receptor signaling pathway	GO:0031295: T-cell costimulation
GO:0007169: Transmembrane receptor protein tyrosine kinase signaling pathway	GO:0007169: Transmembrane receptor protein tyrosine kinase signaling pathway
GO:0042110: T-cell activation	GO:0050852: T-cell receptor signaling pathway
hsa04650: Natural killer cell-mediated cytotoxicity	GO:0042113: B-cell activation
GO:0002250: Adaptive immune response	GO:0050853: B-cell receptor signaling pathway
GO:0042102: Positive regulation of T-cell proliferation	hsa04660: T-cell receptor signaling pathway
hsa04640: Hematopoietic cell lineage	GO:0045060: Negative thymic T-cell selection
GO:0050850: Positive regulation of calcium-mediated signaling	GO:0045086: Positive regulation of interleukin-2 biosynthetic process
hsa04514: CAMs	GO:0070098: Chemokine-mediated signaling pathway
GO:0045060: Negative thymic T-cell selection	GO:0045087: Innate immune response
GO:0045086: Positive regulation of interleukin-2 biosynthetic process	hsa04612: Antigen processing and presentation
GO:0030217: T-cell differentiation	GO:0046641: Positive regulation of alpha-beta-T-cell proliferation
hsa05332: Graft-versus-host disease	hsa04662: B-cell receptor signaling pathway
hsa05330: Allograft rejection	GO:0006954: Inflammatory response
hsa04940: Type I diabetes mellitus	hsa04650: Natural killer cell-mediated cytotoxicity
hsa04612: Antigen processing and presentation	hsa04514: CAMs
hsa04060: Cytokine-cytokine receptor interaction	hsa04060: Cytokine-cytokine receptor interaction
	GO:0006959: Humoral immune response
	GO:0050850: Positive regulation of calcium-mediated signaling
	GO:0042102: Positive regulation of T-cell proliferation
	GO:0071345: Cellular response to cytokine stimulus
	GO:0031529: Ruffle organization
	GO:0006935: Chemotaxis

*: Pathways commonly downregulated in both comparisons are given in italics; GO: Gene ontology; MI: Mild infection; CI: Critical infection; NCI: SARS-CoV-2-negative control individuals; CAMs: Cell adhesion molecules.

**Figure 2.**
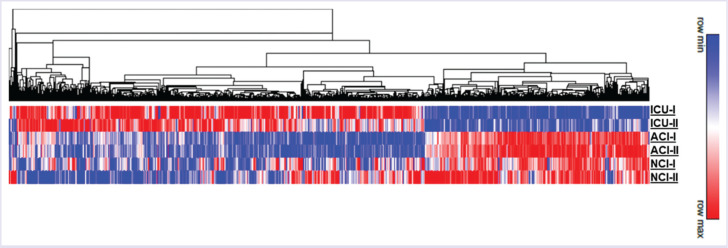
Heat map of differential gene expression among CI, MI, and NCI whole-blood samples. The graphical display is based on a cutoff log2-fold change value of 2.

When genes differentially upregulated in whole blood of CI patients were examined, 354 and 600 genes were found to be upregulated compared to NCI and MI, respectively (Table 2), with 273 upregulated genes common in both comparisons (Fig. 1b). The results of the GO enrichment analysis displayed six pathways commonly upregulated in both comparative conditions (Table 3). These pathways included genes that function in inflammatory response and inflammatory cytokine release (Table 3). Strikingly, platelet activation (GO:0030168) and platelet degranulation (GO:0002576) pathways were exclusively upregulated in CI patients compared to MI but not to NCI. Heat map of differential expression observed among CI, NCI and MI blood samples is provided as Figure 2.

**Table 3.** The comparison of GO pathways upregulated in CI, MI, and NCI patients

**GO pathways upregulated in CI patients**	
CI versus NCI*	CI versus MI*
GO:0045087: Innate immune response	GO:0006953: Acute-phase response
GO:0006954: Inflammatory response	GO:0071222: Cellular response to lipopolysaccharide
GO:0006915: Apoptotic process	GO:0002576: Platelet degranulation
GO:0071222: Cellular response to lipopolysaccharide	GO:0006954: Inflammatory response
GO:0061621: Canonical glycolysis	GO:0045087: Innate immune response
GO:0006953: Acute-phase response	GO:0032720: Negative regulation of tumor necrosis factor production
GO:0050832: Defense response to fungus	GO:0032715: Negative regulation of interleukin-6 production
GO:0042742: Defense response to bacterium	GO:0030168: Platelet activation
GO:0006096: Glycolytic process	
GO:0051092: Positive regulation of NF-kappa B transcription factor activity	
hsa04066: HIF-1 signaling pathway	
hsa05132: Salmonella infection	
hsa04668: TNF signaling pathway	
hsa05140: Leishmaniasis	
hsa05134: Legionellosis	
GO:0032496: Response to lipopolysaccharide	
GO:0032720: Negative regulation of tumor necrosis factor production	
GO:0050900: Leukocyte migration	
GO:0032715: Negative regulation of interleukin-6 production	
GO:0050830: Defense response to Gram-positive bacterium	
hsa04620: Toll-like receptor signaling pathway	
hsa00010: Glycolysis/gluconeogenesis	
GO:0031663: Lipopolysaccharide-mediated signaling pathway	
GO:0006970: Response to osmotic stress	

*: Pathways commonly upregulated in both comparisons are given in italics; GO: Gene ontology; MI: Mild infection; CI: Critical infection; NCI: SARS-CoV-2-negative control individuals.

## Discussion

Differentially expressed genes in CI patients compared to NCI and MI were identified as a result of the study. A log2-fold change value of 2 was used as a cutoff to accept genes as differentially expressed. It is feasible to adjust lower cutoff values, which would result in the identification of a higher number of genes as differentially expressed. However, it is useful to set high stringency statistical analysis conditions to compensate for the relatively narrow sample size in cases where sampling from a high number of individuals is not feasible. Nevertheless, a significantly high number of genes were found to be differentially expressed in CI patients compared to both NCI and MI individuals when differentially expressed genes were determined based on a statistically safe cutoff threshold. GO enrichment analysis determined the up- and down-regulated pathways. We identified 21 GO pathways commonly downregulated in CI patients compared to both NCI and MI. Downregulated 17 GO pathways were directly associated with innate and adaptive immune responses. Three hundred and fifty four and 600 genes were found to be upregulated compared to NCI and MI, respectively. Upregulated six pathways included genes that function in inflammatory response and inflammatory cytokine release.

Immune responses to SARS-CoV-2 infection include tissue barriers, innate and adaptive cells, and mediators. Once the virus infects respiratory epithelial cells, viral peptides are presented through Class I major histocompatibility complex (MHC) proteins to CD8+ cytotoxic T cells, leading to the activation and development of virus-specific effector and memory T cells. In short time, virus is recognized by antigen-presenting cells which present viral peptides to CD4+ T cells through MHC-Class-II molecules, while B cells can directly recognize the virus and interact with CD4+ T cells. Immunoglobulin (Ig) M virus-specific antibody response is observed within the 1^st^ week following symptoms and IgG antibodies follow that, mostly retaining a lifelong immunity [17].

A wide variety of mild, moderate, severe, and rapidly progressive clinical findings are observed in patients with SARS-CoV-2 [18]. SARS-CoV-2 infection is not simply common cold and majority of SARS-CoV-2-infected individuals might have no or mild symptoms. The mortality rate of SARS-CoV-2 is estimated as 3–4%, which is higher than mortality rate (<0.1%) of influenza infection [19]. High prevalence of SARS-CoV-2 plasma viral load was found related to increased respiratory disease severity, low lymphocyte counts, and increased inflammation markers such as C-reactive protein and interleukin (IL)-6 [20]. The cytokine storm in severe COVID-19 is characterized by Pedersen et al. [19] and decreased interferon (IFN)-γ expression in CD4+ T cells, lymphopenia (in CD4+ and CD8+ T cells), and increased cytokine levels (IL-6, IL-10, and TNF-α) were found to be associated with severe COVID-19, likely through increased pulmonary pathology, T-cell depletion, and CD4+ T cell dysfunction. Another study showed that the NOD-like receptor family, pyrin domain-containing 3 inflammasome in peripheral blood mononuclear cells and tissues of postmortem patients is activated in response to SARS-CoV-2 infection. Higher levels of Casp1p20 and IL-18 which are inflammasome-derived products in the sera were found associated with COVID-19 severity and poor clinical outcome, including IL-6 and lactate dehydrogenase [21]. Furthermore, viral RNA is sensed by toll-like receptor TLR3, TLR7, TLR8, and TLR9, activating the NF-κB pathway and pro-inflammatory cytokines, initiating virus-induced inflammation. Host immune signaling proteins are targeted by SARS-CoV-2 viral proteins. For instance, IFN pathway is targeted by Nsp13, Nsp15, and open reading frame ORF9b [17]. SARS-CoV infection was indicated a depressed innate immune response. with decreased expression of genes related to toll-like receptor and IL signaling [22–24]. As shown in Table 2, gene expression of innate and adaptive immune response is downregulated in critical ill patients. Furthermore, Mick et al. [8] were represented gene expression in the upper airway tissue and showed that gene expression of innate immune response diminished compared to other virus infections. According to our results, majority of downregulated GO pathways in CI (17 pathways) were directly associated with innate and adaptive immune responses, a result that is attributed to the corticosteroid treatment-associated downregulation of systemic and pulmonary inflammation. For instance, CD4, CD6, CD3g, TLR7, and IL32 were common genes downregulated in CI versus NCI or MI patients. Remaining downregulated GO pathways in CI versus NCI and CI versus MI were also associated with the immune system responses, including pathways such as innate immune response (GO:0045087), inflammatory response (GO:0006954), B-cell activation (GO:0042113), B-cell receptor signaling (GO:0050853), T-cell differentiation (GO:0030217), and chemokine-mediated signaling (GO:0070098). Some of the associated genes were TLR7, IL-27 receptor subunit alpha, IL-5 receptor subunit alpha, NLR family CARD domain-containing protein 3, IL-32, T-cell receptor gamma variable 2, CD molecules (CD2, CD4, CD6, CD7, CD22, CD74, etc.), Ig superfamily member 8, and B and T lymphocyte associated which are given in Table 2. Our results show that the expression of immune elements that should be activated by SARS-CoV-2 infection is suppressed in severe patients. On the other hand, when we examine genes that are upregulated, 354 and 600 genes were found to be upregulated compared to NCI and MI, respectively. The results of the GO enrichment analysis displayed six pathways including genes that function in inflammatory response and inflammatory cytokine release. TLR4, TLR8, IL4 receptor, IL-1 receptor-like 1, lactate dehydrogenase A, NLR family CARD domain-containing 4, CD24, CD63, and CD177 were upregulated in CI versus NCI and/or MI (Table 3). The fact that some immune elements are upregulated, and some are downregulated may indicate an impaired immune response in severe patients. Interestingly, it was observed that some members of the matrix metalloproteinase (MMP) family (MMP-8-9 and 25) had upregulated expression levels in CI patients which may indicate the breakdown of extracellular matrix in intensive care patients. In addition, human phenotype ontology analysis of the entire set of upregulated genes in CI patients versus MI displayed “abnormal thrombosis” as the top human phenotype (HP: 0001977) associated with the gene set. The contemporary activation of immune, inflammatory, and coagulation pathways is consistent with the concept of immunothrombosis [2]. These two processes are initially triggered by a diffuse endothelial dysfunction induced by SARS-CoV-2 through ACE-2 receptors and the transmembrane protease serine 2 that are currently recognized as specific sites by which the virus enters into the vascular system. One of the worst features of COVID-19 is a severe coagulopathy with an increased risk of thromboembolic complications and an incidence of venous thromboembolism (VTE) of about 25%. In critically ill patients, VTE incidence was 25% and higher than the non-COVID patients [25]. Furthermore, arterial thrombosis is not rare (3.7%) [26]. As shown in Table 3 genes that function in inflammatory response, inflammatory cytokine release, platelet activation, and platelet degranulation were upregulated. These genetic regulations may explain the immunothrombosis which common cause of morbidity.

Thrombosis is reported to contribute significantly to the severity of COVID-19 symptoms [27–30]. Our results provide evidence at the gene expression level for the role of thrombosis in defining the severity of the disease. Yet, it remains to be determined whether platelet hyperactivation is the cause or result of severe COVID-19 symptoms. Further evidence can be collected by targeted expression analysis of genes in platelet activation/degranulation pathways over the course of the disease and treatment. Notably, the expression of the ACE-2 gene encoding the putative cell entry receptor of SARS-CoV-2 was found to be upregulated in whole blood of CI patients with respect to MI (Table 3) but ACE2 expression level did not differ from NCI. Indeed, it is proposed that SARS-CoV-2 may directly infect and activate megakaryocytes and platelets [30, 31]. Yet, the mechanism of interaction and direct activation remains controversial with contrasting reports on the presence of ACE2 protein expression on platelets. ACE2-mediated or ACE2-independent entry of SARS-CoV-2 to platelets through CD147 and CD26 as the binding partners is both proposed for direct SARS-CoV-2 activation of platelets, leading to thrombosis [28]. According to our results, ACE2 expression in whole blood was elevated in CI patients with respect to MI but CD147 and CD26 were not among the differentially expressed genes in CI patients.

Limitations of this study are limited number of patients and that the study design was cross sectional. Gene expression regulation is actually highly dynamic and gene expression profiles of both CI and MI patients may change during the period of COVID-19 infection in both groups. Following changes in gene expression, patterns with periodic recurrent tests can be useful to reveal infection phase-dependent differences in gene expression between CI and MI patients. Age differential may affect the observed gene expression differences, on the other hand, the fact that majority of downregulated genes in CI patients belong to pathways associated with immune responses imply the effect of glucocorticoid therapy on gene expression. Yet, regardless of age and differences in therapeutic applications, the pool of differentially expressed genes detected in this study unquestionably includes genes that are associated with severe disease symptoms. It is important to note that Favipravir was administered to both CI and NCI patients, which also would cause changes in gene expression regulation. Yet, the gene expression profile of CI patients deviates more significantly from the MI patients compared to NCI individuals. Thus, gene expression differences observed in the present work result from not only differential drug treatments but also differential disease severity in CI and MI individuals.

Overall, it is noteworthy that the transcriptional profile of CI patients deviates more significantly from that of MI in terms of the number of differentially expressed genes. These results imply that genotypic differences may account for the severity of SARS-CoV-2 infection responses. In addition, upregulated blood ACE2 expression in CI patients was observed only compared to MI, supporting the putative role of genotypic differences in disease severity. To resolve genotype – disease susceptibility associations, combined studies that involve both genomic and transcriptomic analyses are essential.

### Conclusion

Differential transcriptomic analysis highlights the necessity of genomic association studies, therefore, further work involving the genotyping of large groups of asymptomatic and symptomatic individuals as well as transcriptomic analyses would help determine genotypic associations with the severity of COVID-19 symptoms.
